# Supplementary material for: Resistance to targeted therapies as a multifactorial, gradual adaptation to inhibitor specific selective pressures
Source: Nat Commun. 2020 May 14;11:2393. doi: 10.1038/s41467-020-16212-w (PMC7224215; doi:10.1038/s41467-020-16212-w)
Supplement: Supplementary file 1 — Supplementary Figures and Methods [file 41467_2020_16212_MOESM1_ESM.pdf]

Supplementary Figures

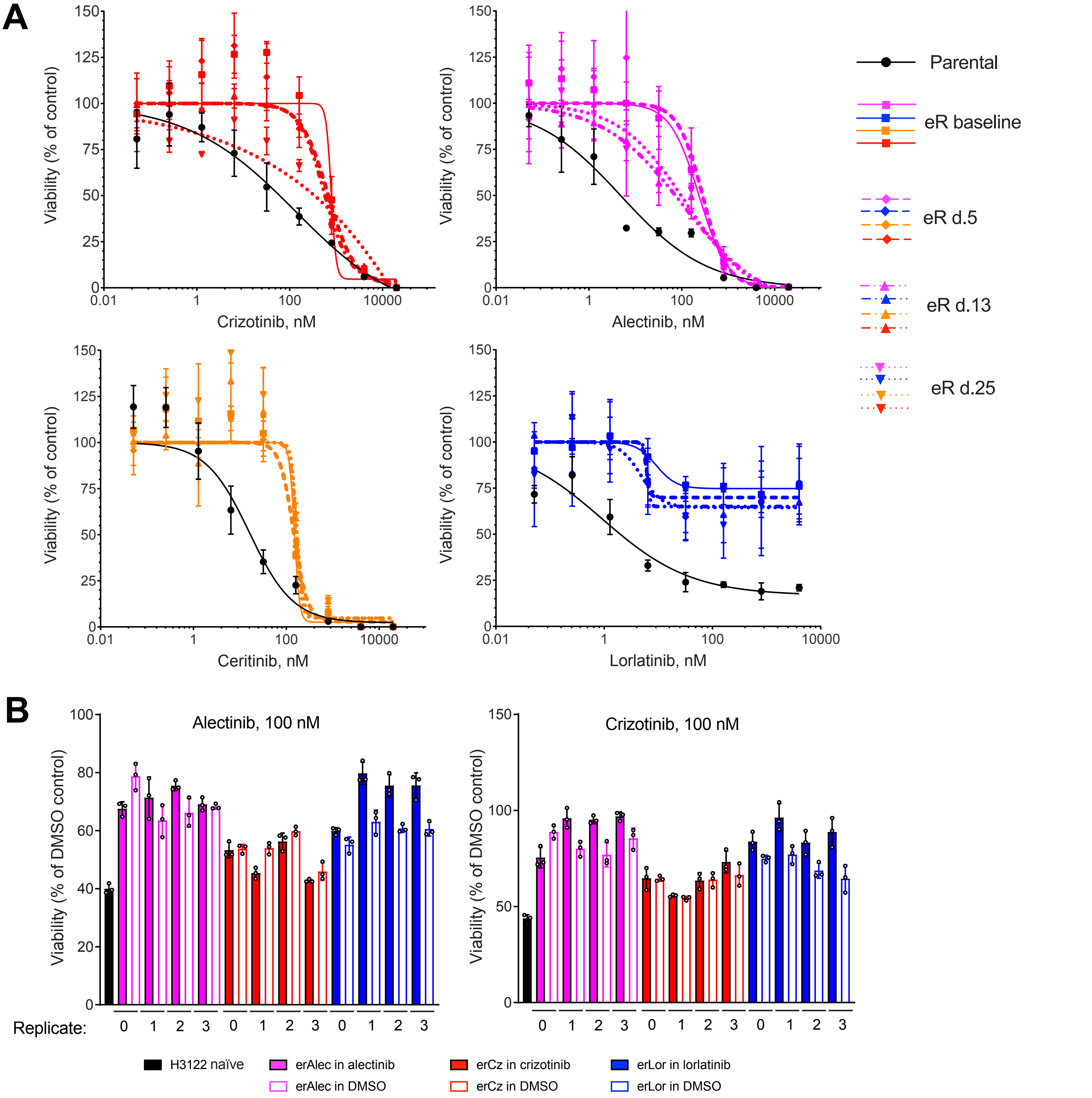

Supplementary Figure 1. **Heritability of resistant phenotypes.**
A. Stability of ALK-TKI resistance is assessed by analysis of ALK TKIs sensitivity of indicated erALK TKI cell lines cultured in the absence of the drugs for indicated periods of time using Cell Titer Glo assay. Mean +/- SD of experimental triplicates (separate wells) are shown. **B**. Stability of ALK TKI resistance of independently derived cell lines, assessed by sensitivity to 100 nM alectinib and crizotinib following 4 weeks of culture in the absence of the drug. Notation of individual cell lines is identical to that used in Fig. 1F. Mean +/- SD of experimental replicates are shown.

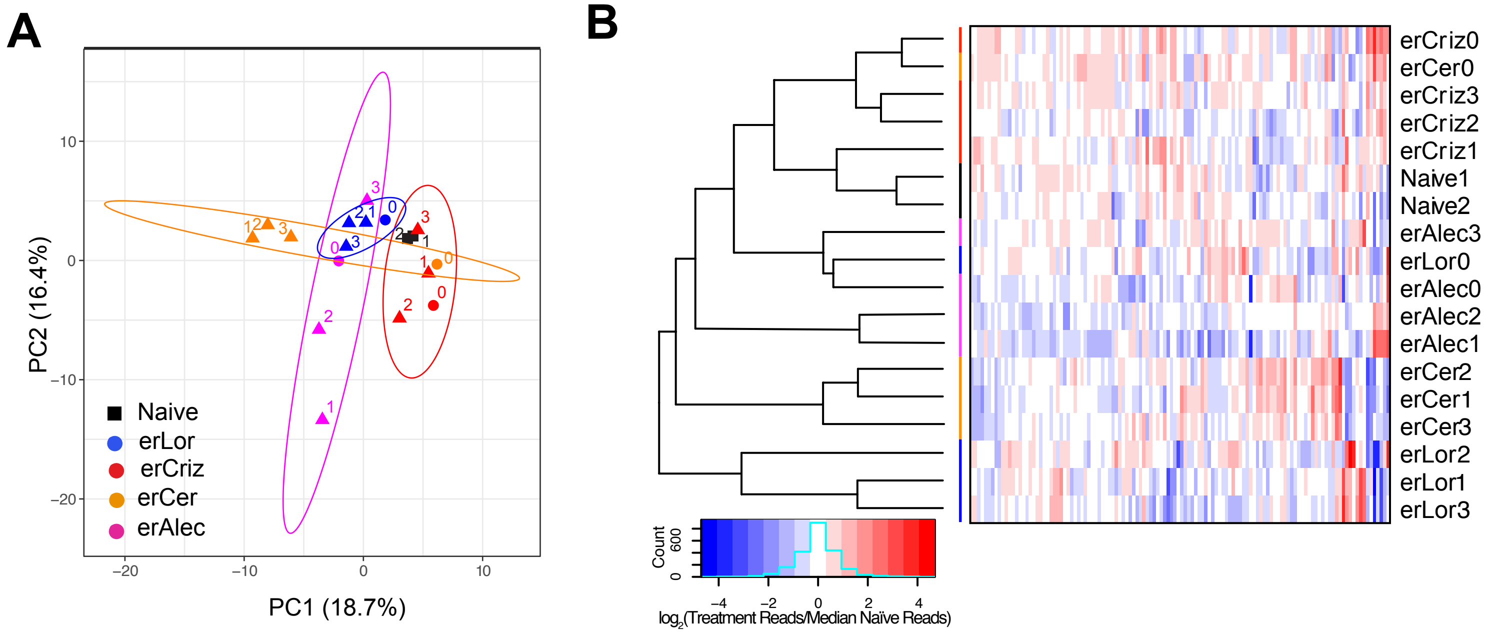

Supplementary Figure 2. **Nanostring profiling of erALK-TKI lines**.
PCA analysis (**A**) and hierarchical clustering analysis (**B**) for the differentially expressed Nanostring nCounter GX human cancer reference panel transcripts of the indicated samples. Notation of individual cell lines is identical to that used in Fig. 1F.

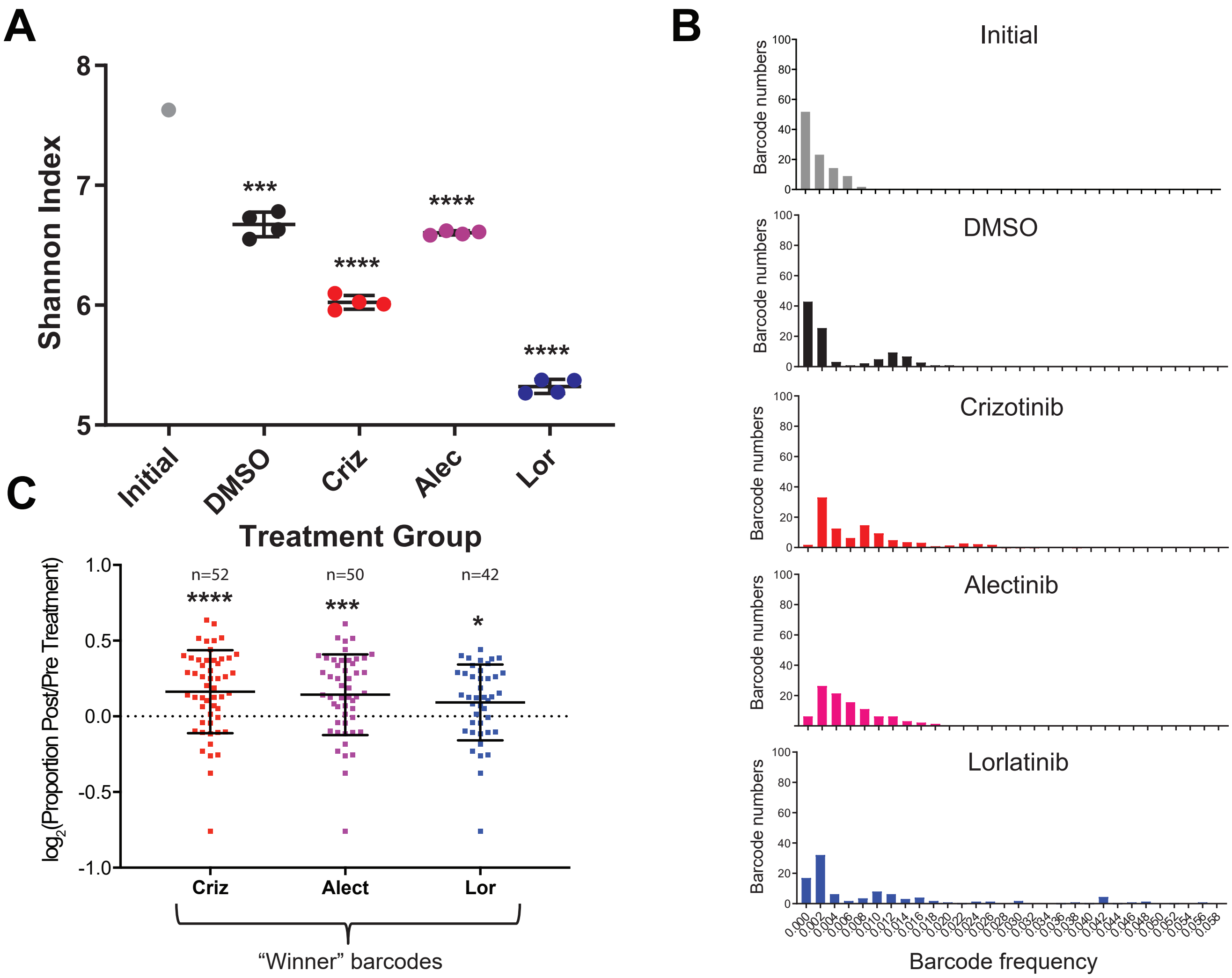

Supplementary Figure 3. Subpopulation tracing analyses.
A. Shannon diversity of all barcodes after treatment. Error bars show standard devivation. ***, **** represent p<0.001 and p<0.0001 in a one-sample, two-tailed, t-test comparing Shannon diversity post-selection to the baseline sample. Mean +/- SD of experimental replicates are shown. n=1 for pre-treatment, n=4 for all others, representing separate dishes. **B.** Frequency distributions of barcodes. Only barcodes that exceed the most common pre-treatment barcode in at least one sample are shown. Replicates in the same treatment are merged, the mean frequency across all replicates is plotted. **C.** Frequency of barcodes under DMSO control conditions. Only barcodes exceeding the highest frequency in the initial mixture are shown. Mean +/- SD of experimental replicates are shown. *, *** and **** represent p<0.05, p<0.001 and p<0.0001 in a one sample, two-tailed, t-test comparing means to zero, n represents separate barcodes.

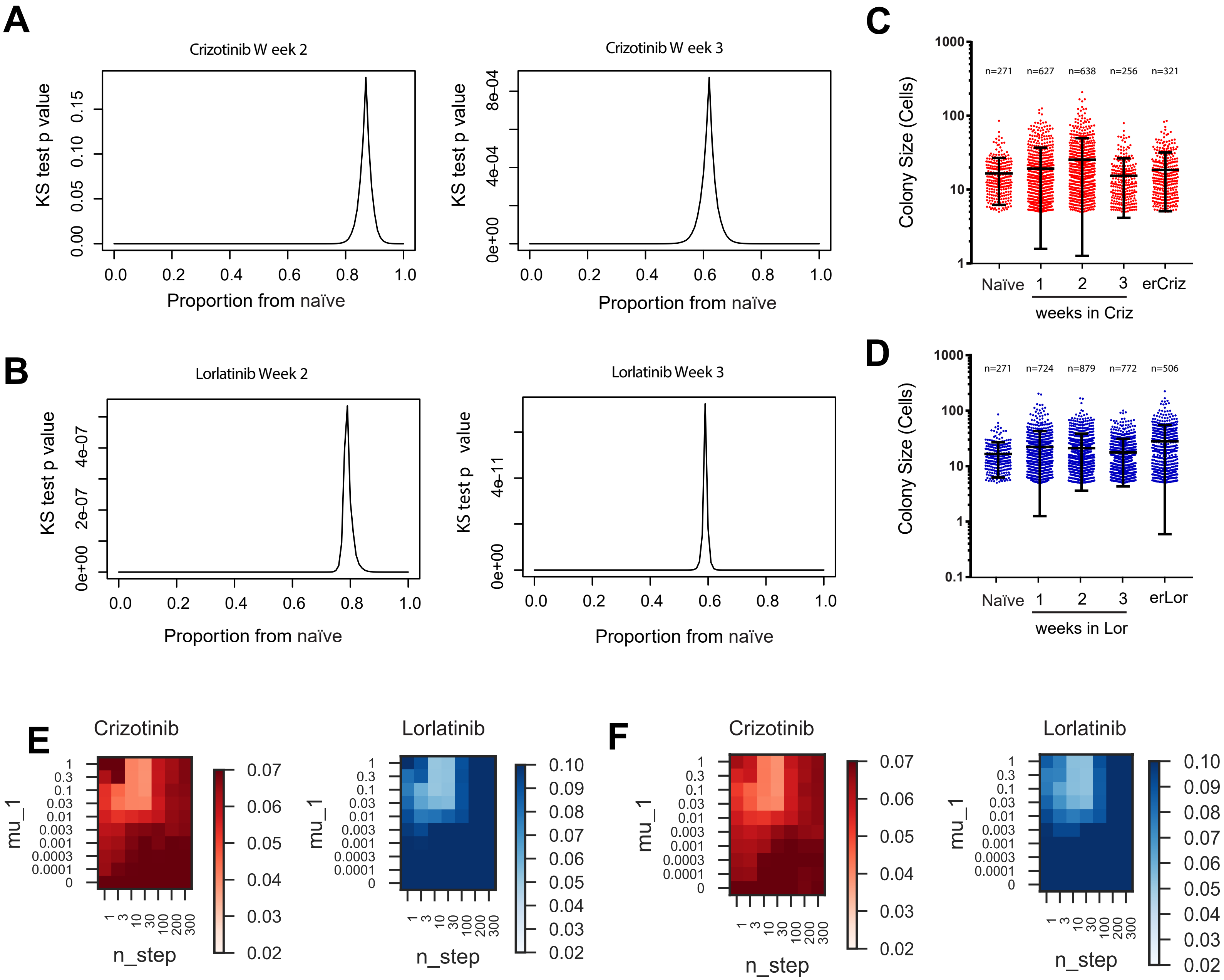

Supplementary Figrue 4. Graduality of evolution of erALK-TKI.
A, B. Kolmogorov-Smirnov test estimates for likelihood that the distribution at an intermediate time point can be explained by combined sampling from DT cells (naïve cell colony distribution) and erALK-TKI cells, for growth in crizotinib (A) and in lorlatinib (B). **C-D.** DMSO control colony size distribution of treatment naïve H3122 cells, cultured in the presence of 0.5 μM crizotinib (C) or lorlatinib (D) for the indicated duration of time prior to the clonogenic assay. Mean +/- SD of experimental replicates are shown, data points represent individual colonies. **E, F.** Kullback–Leibler divergence-based comparison of the experimental data with the outcomes of simulations, covering parameter spaces for the indicated mutation probabilities and number of mutational steps, with the inclusion of death probability (E) and an additional inclusion of mutations that cause bi-directional fitness changes (F).

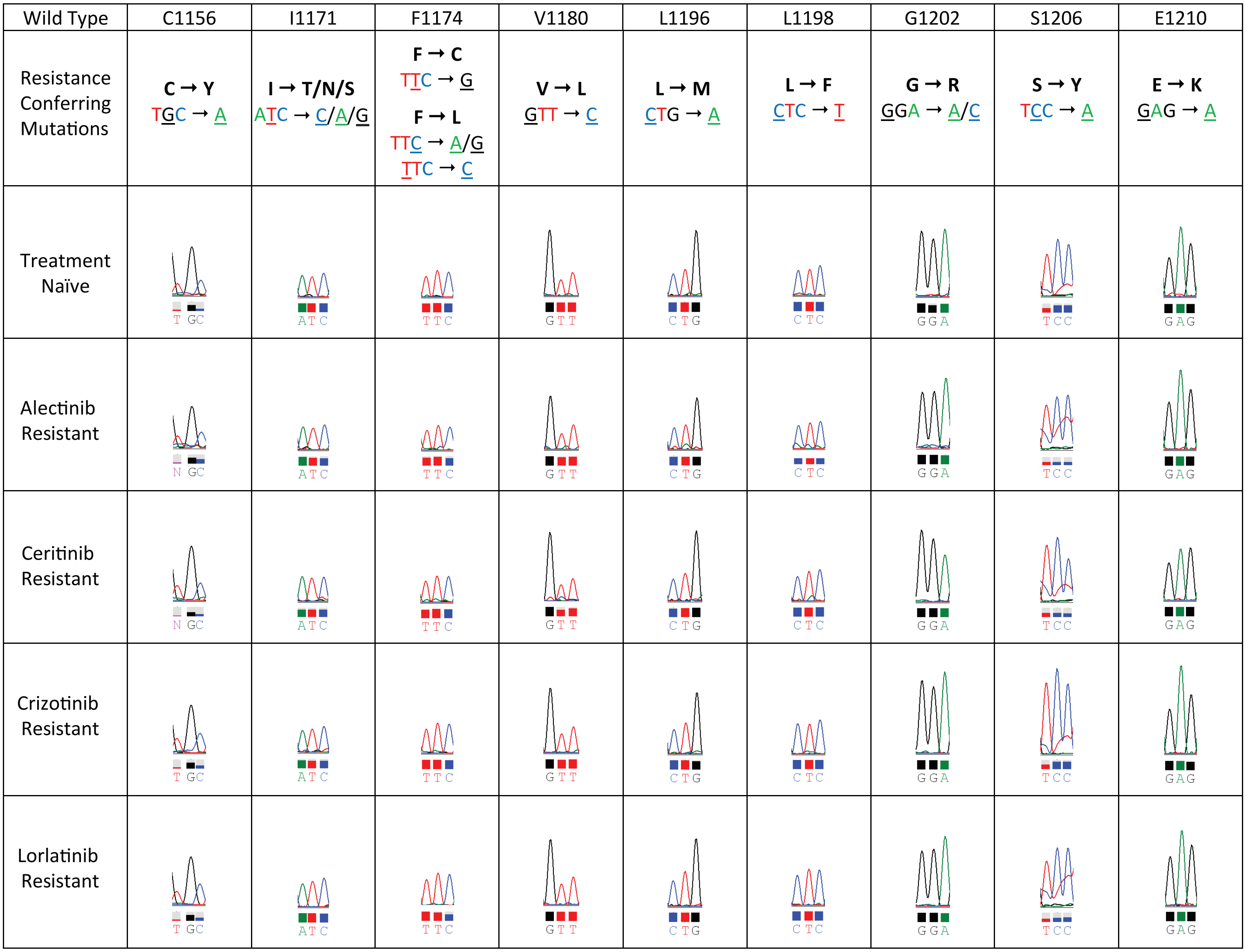

Supplementary Figure 5. Sanger Sequencing of Select ALK Point Mutation Sites.
Sanger sequencing traces for the indicated resistance-associated EML4-ALK hotspot mutations.

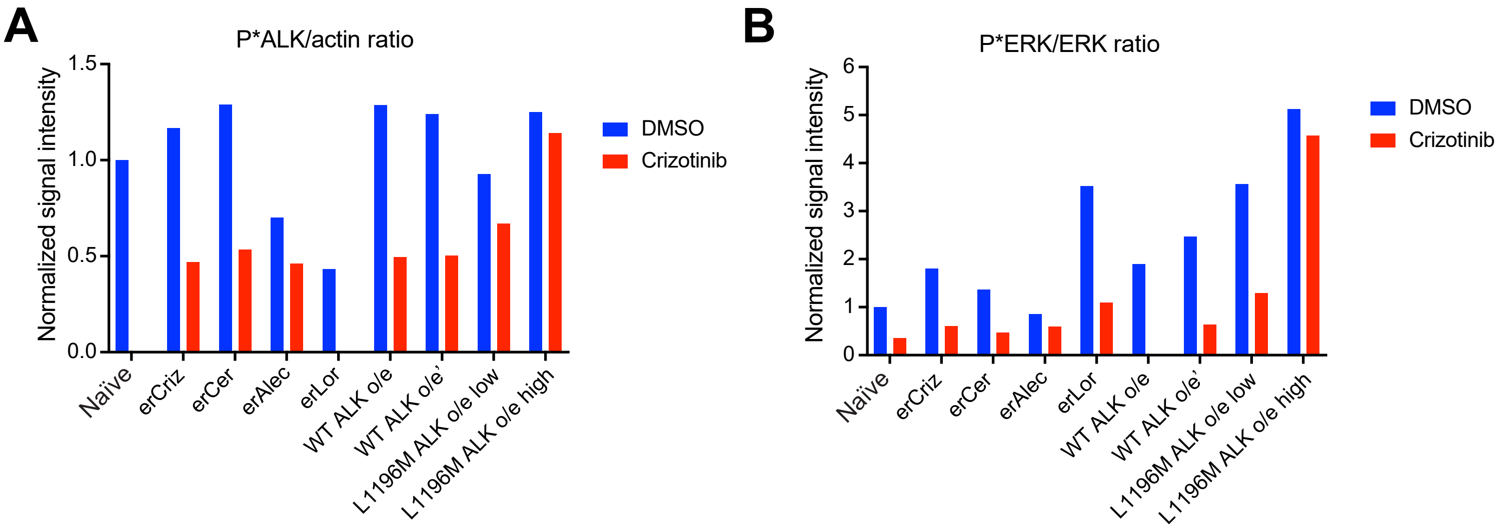

Supplementary Figure 6. Quantitation of immunoblot data from Fig. 4E.
A. Phospho-EML4-ALK/actin ratio. To account for changes in EML4-ALK expression in overexpressing cell lines, the data is normalized to EML4-ALK/actin ratio in the DMSO control naïve cells. **B.** Phosphorylated to total ERK ratio, normalized to the ratio in the DMSO control naïve cells.

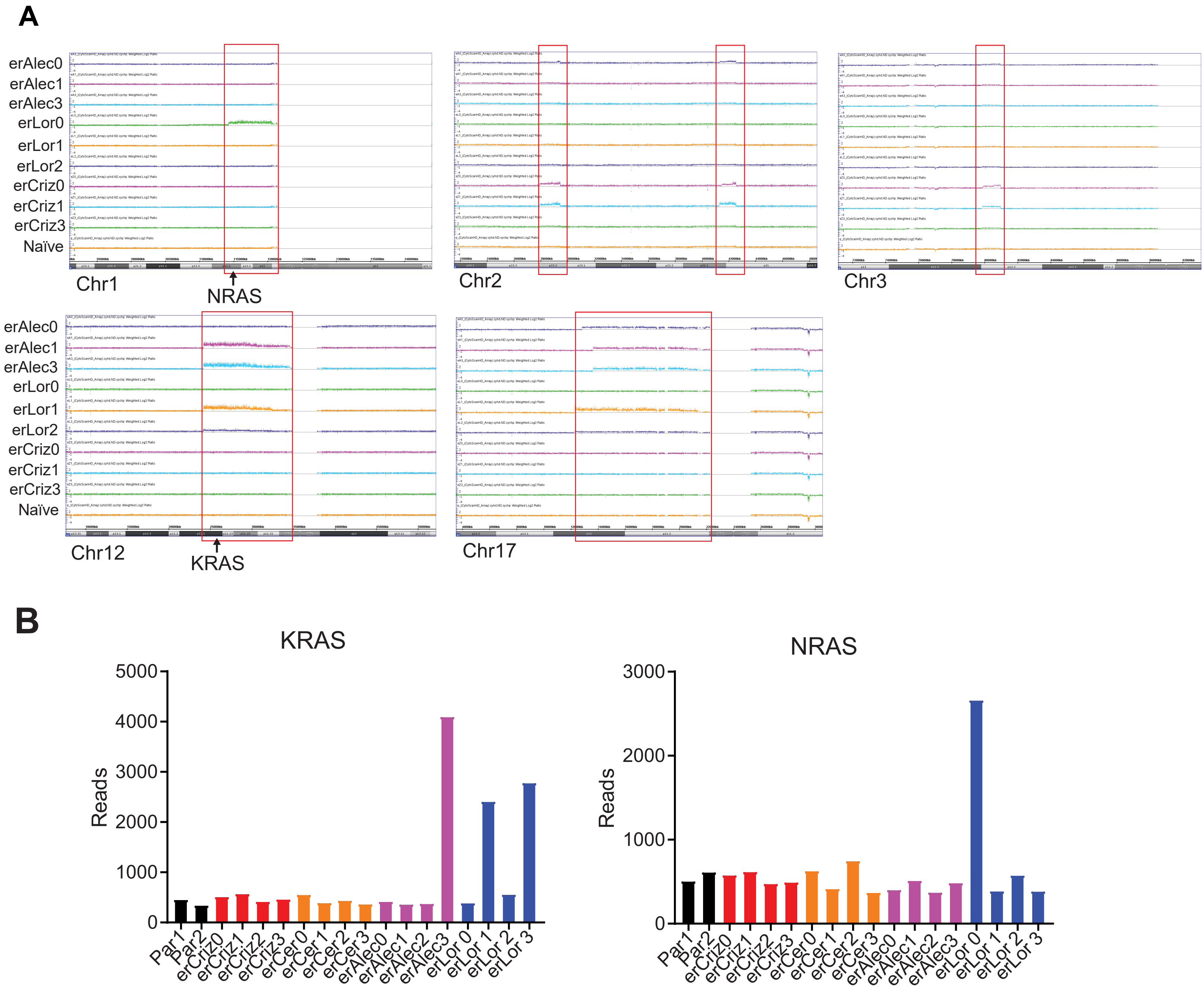

Supplemental Figure 7. CNV analyses of erALK-TKIs.

A. Weighted log2 ratio in regions selected based on differences between parental H3122 and at least one resistant line, differences are highlighted by red boxes. The genomic locations of KRAS and NRAS are highlighted. **B.** KRAS and NRAS reads obtained from the NanoString expression data.

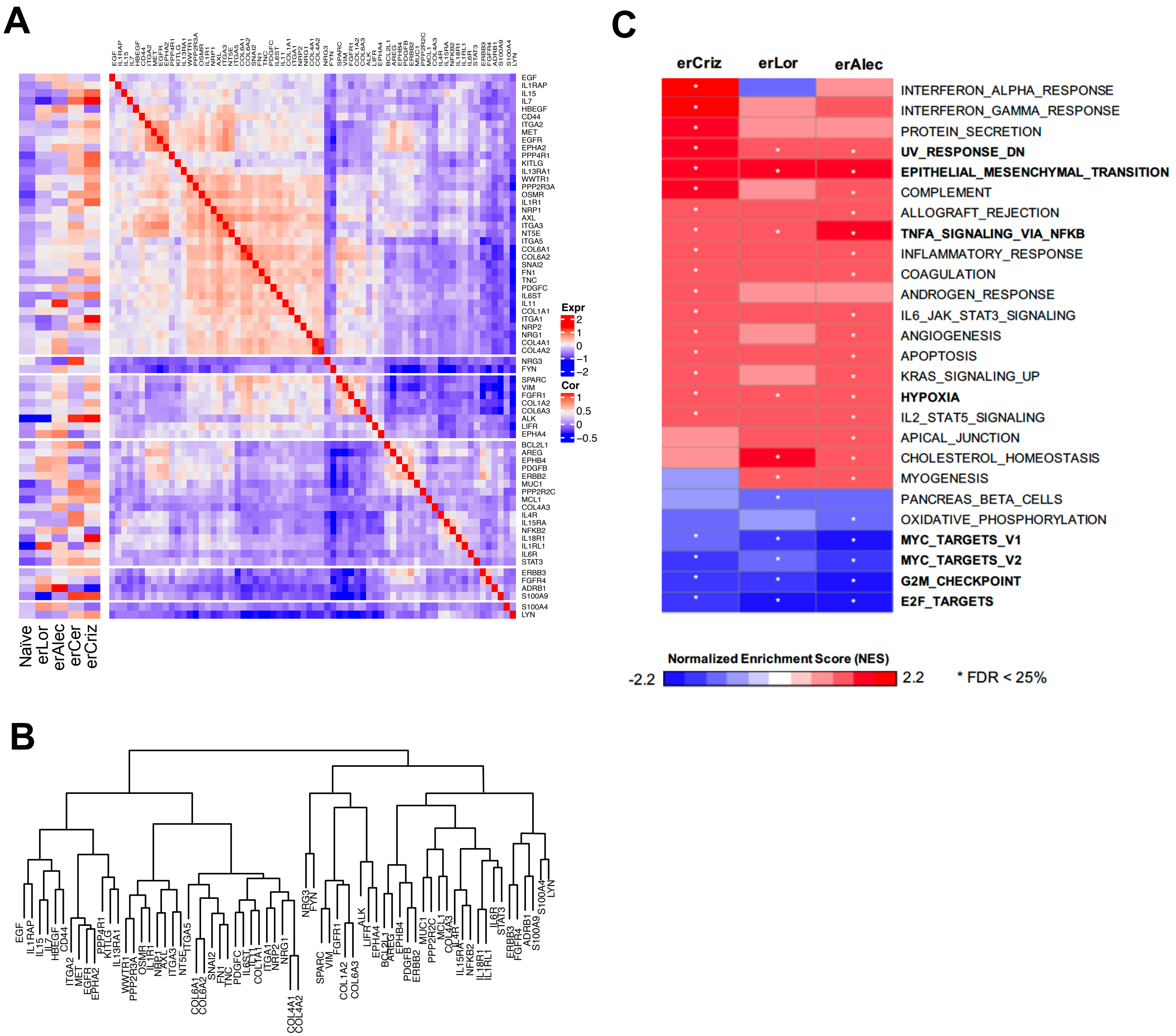

Supplementary Figure 8. Transcriptomics changes in erALK-TKI cells.
A. Correlation analysis for co-expression of resistance-associated genes, upregulated in erALK-TKI cell lines. **B**. Hierarchical clustering dendrogram for the indicated individual overexpressed genes. **C**. Gene set enrichment analyses results from three acquired resistant cell lines were plotted as a heatmap using Normalized Enrichment Score (NES), gene sets with FDR ≤0.25 is indicated by an asterisk (*).

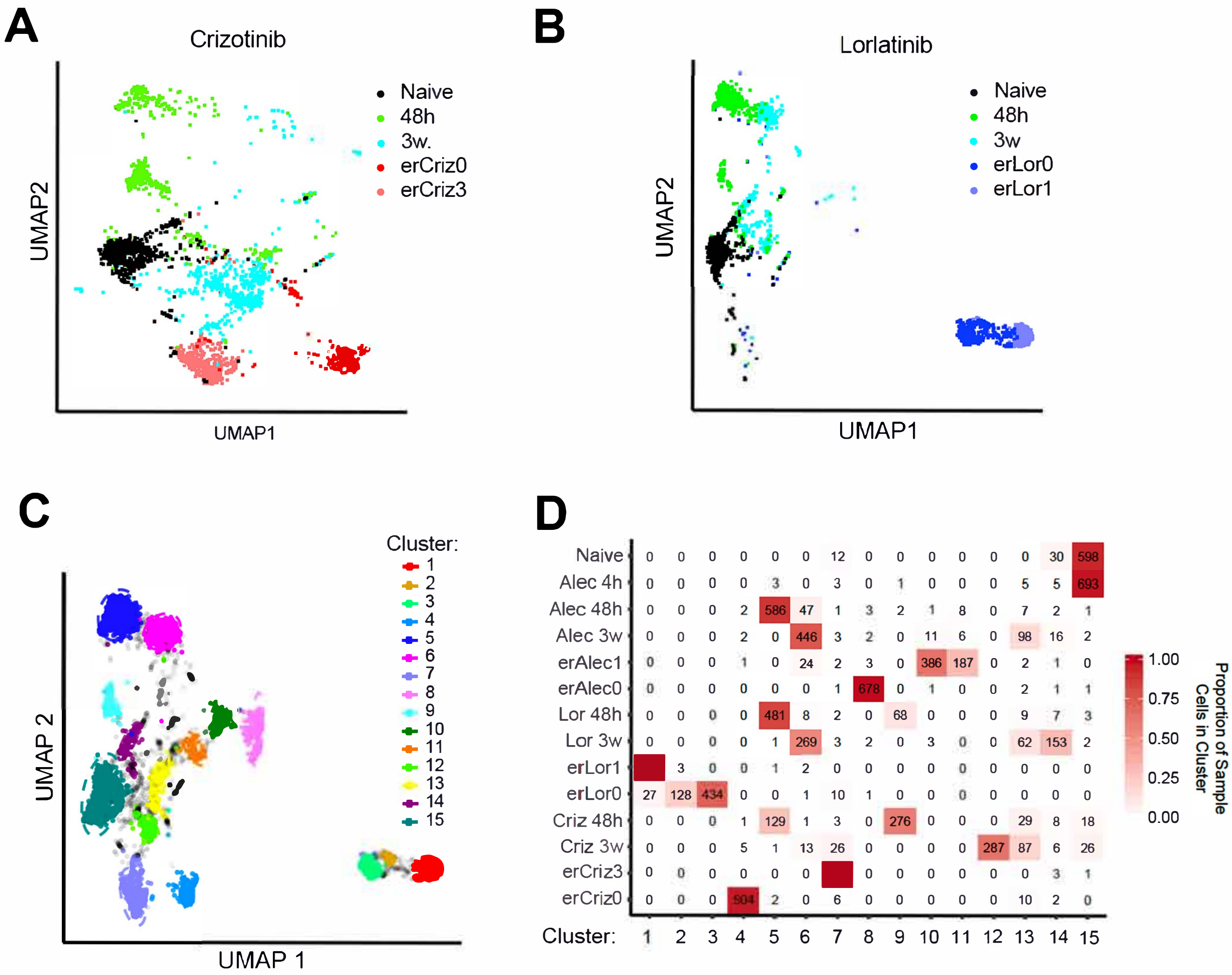

Supplementary Figure 9. Phenotypic evolution in response to ALK-TKIs.

A, B. Single cell RNA seq based UMAP analysis of phenotypes at indicated time points post initiation of crizotinib (**A**) and lorlatinib (**B**) treatment, and in the indicated erALK TKI cell lines (one outlier cell lies outside the axes). **C**. Cluster assignment for the UMAP of single cell transcriptomics data from all samples shown in Fig. 1G, 5B, S9 A, B. Gray cells are unassigned. Ellipsoids show 95% confidence intervals. **D**. Heatmap reporting the number of cells in each cluster and sample. Shading is based on the proportion of cells in a sample that are in a specific cluster. Cluster-0 are unassigned cells.

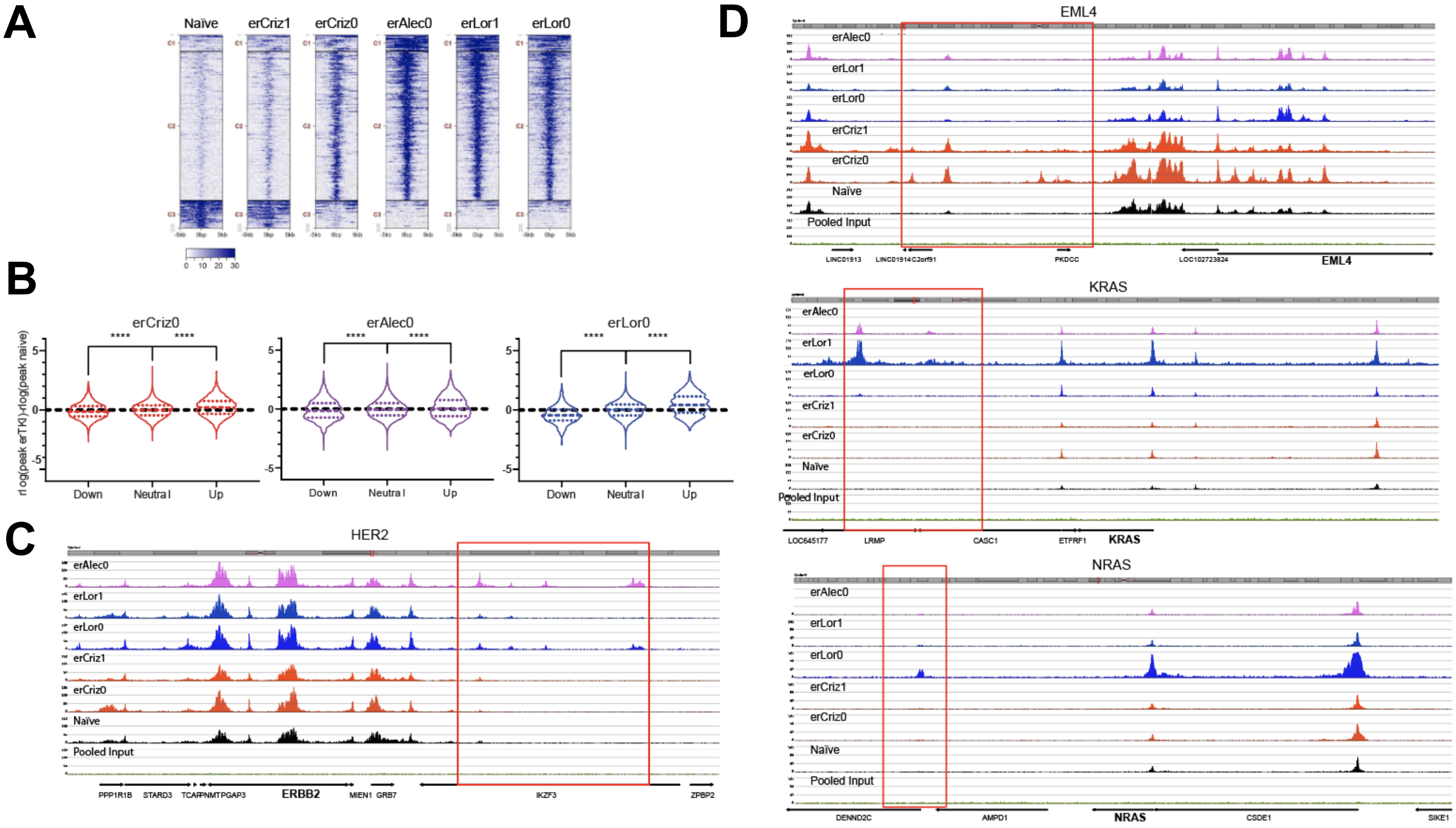

Supplementary Figure 10. H3K27ac ChiP-Seq analyses

A. Clustering of peaks with differential H3K27 acetylation in the indicated cell lines. **B**. Changes in H3K27 acetylation in differentially expressed genes. Black dashed line represents zero, colored dashed lines are medians, colored dotted lines represent quartiles. **** represents p<0.0001 in a Kruskal-Wallis test. **C**. Elevated expression of HER2 is associated with formation of novel H3K27 peaks in the gene's vicinity. **D**. Changes in H3K27 acetylation in the vicinity of the indicated genes.

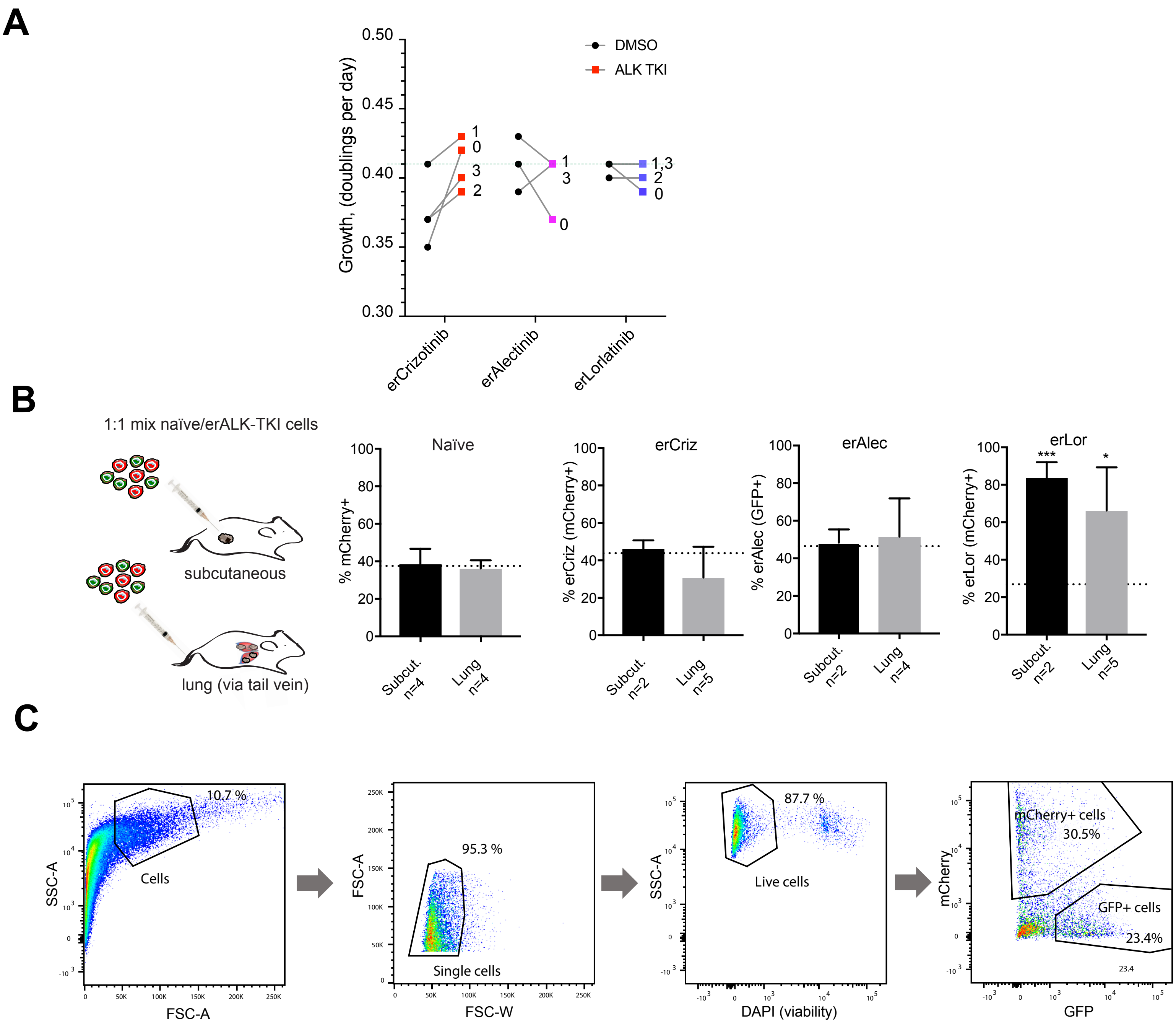

Supplemental Figure 11. Baseline fitness of erALK-TKI cells.

A. Net growth rates of the indicated erALK-TKI cell line in the presence and absence of 0.5 µM of the indicated ALK inhibitors, determined by counting cells over 4 weekly passages. Dashed line represents growth rates of therapy naïve cells cultured under DMSO control. **B.** Flow cytometry-based quantitation of the frequency of the indicated erALK TKI cells (evolved through dose escalation), mixed with differentially labelled therapy naïve H3122 cells and transplanted subcutaneously or orthotopically, via tail vein injection. Y axes show percentages of the indicated fluorescent sub-population as a fraction of all of fluorescent cells. Dashed lines represent frequency of cells in the initial mixtures. * and *** represent $p<0.05$ and $p<0.001$ respectively in a one-sample, two-tailed, t-test comparing means to the initial proportion of cells. Measure of center shows mean. Error bars show standard deviation. Replicates represent separate tumors subcutaneous tumors and, for lungs, separate mice. **C.** Gating strategy is shown for an example (erAlec-GFP/Naïve-mCherry subcutaneous transplant) for the analysis of data presented in B.

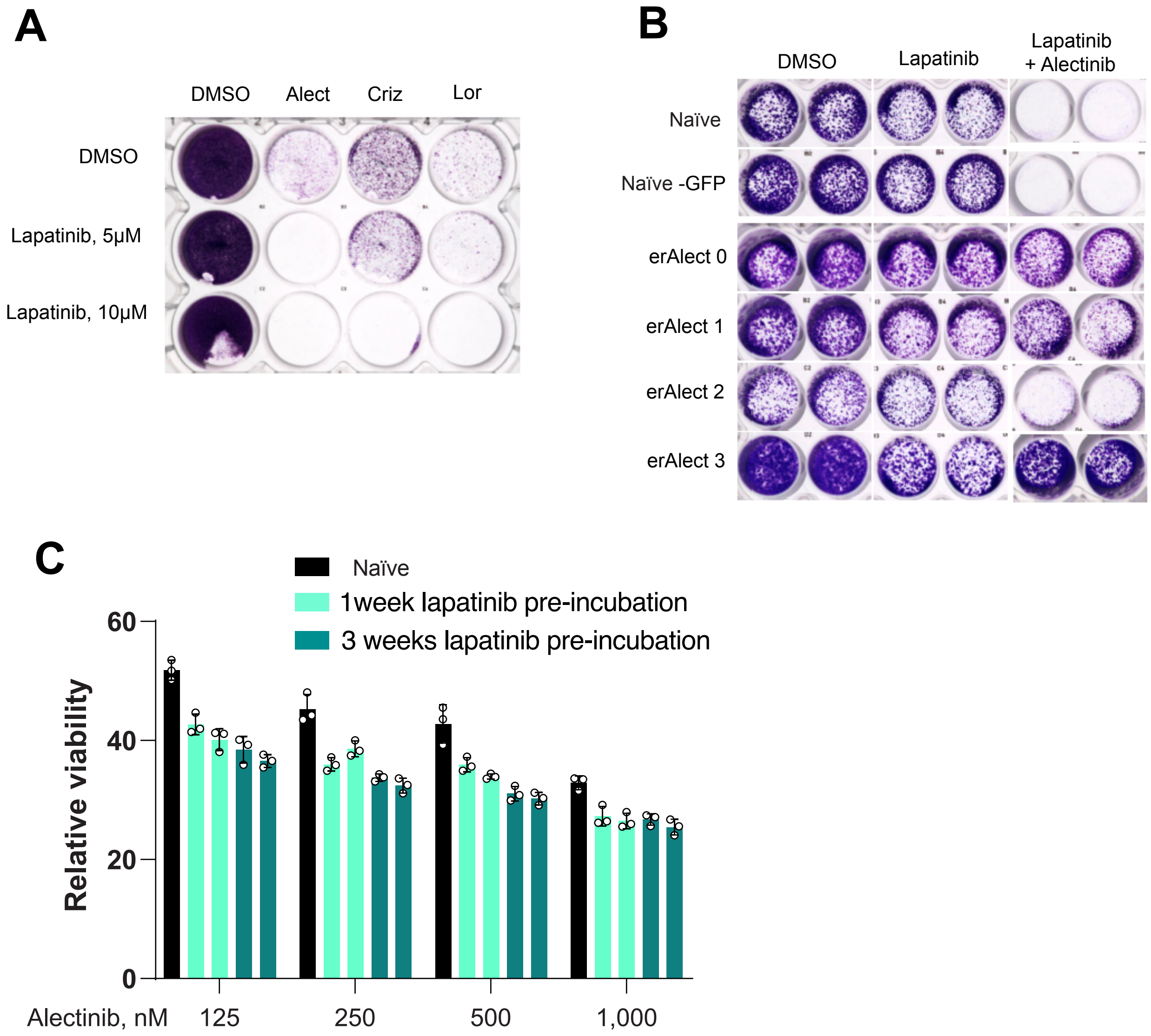

Supplementary Figure 12. Collateral sensitivity to lapatinib.
A. Drug naïve H3122 cells were cultured in the presence of the indicated ALK TKI (0.5 μM) or DMSO control with or without indicated concentrations of lapatinib for 6 weeks, then stained with crystal violet. **B.** Sensitivity of treatment naïve and er-ALK TKI to lapatinib mono or combined therapy, determined after 7 days of treatment by staining with crystal violet. **C.** Alectinib sensitivity of H3122 cells, cultured in the presence of 10 μM lapatinib for 1 and 3 weeks, was compared to the sensitivity of naïve H3122 cells using Cell Titer Glo viability assay. Mean +/- SD of experimental replicates (separate wells) are shown.

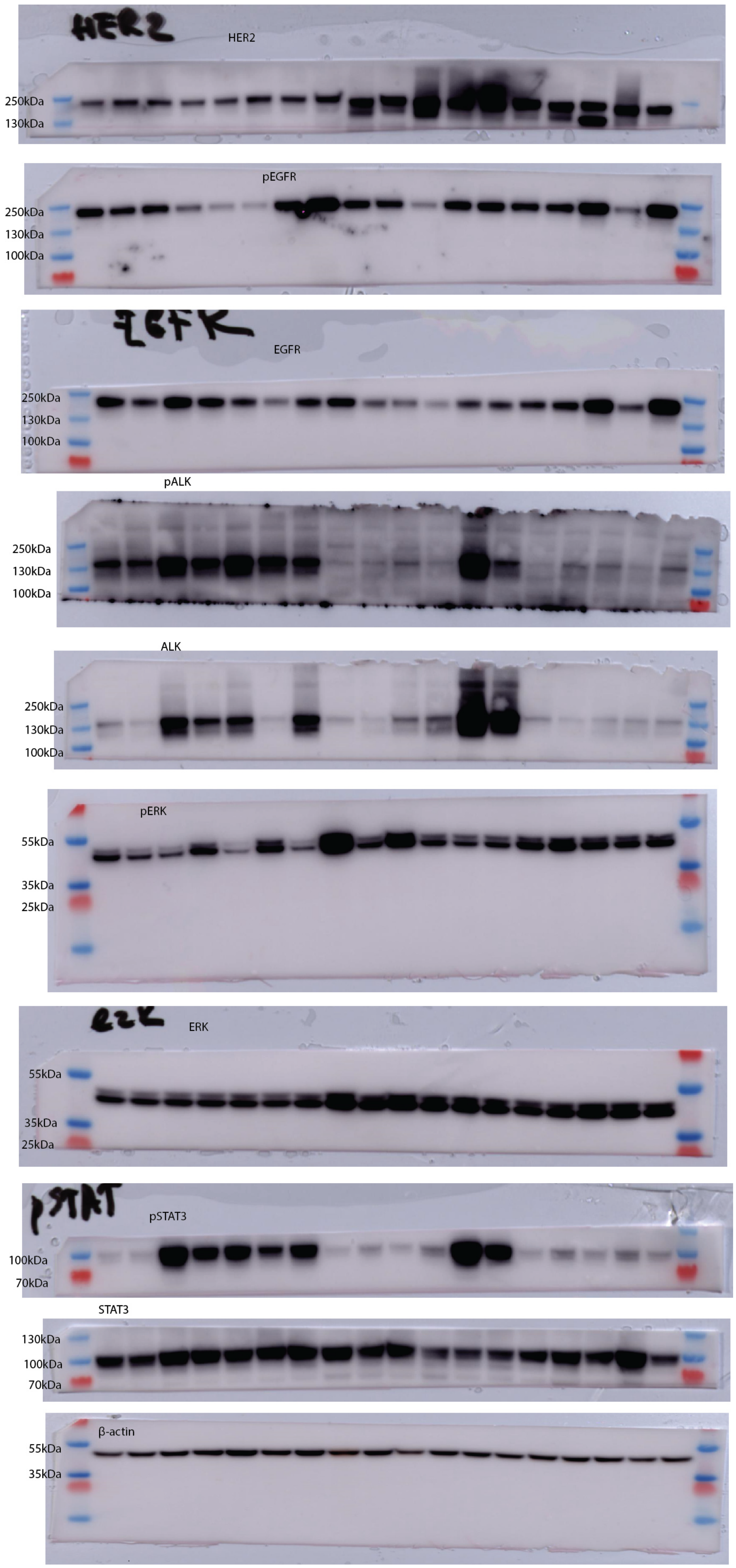

Supplementary Figure 13. Original i **mages for Fig. 1F.**

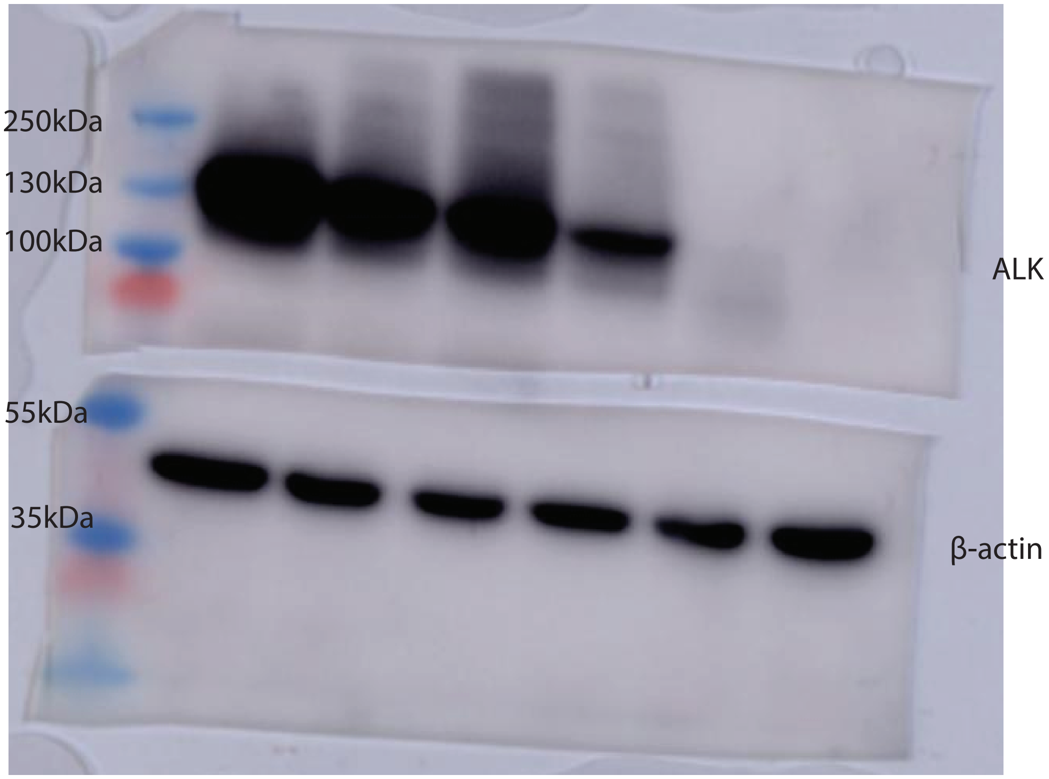

Supplementary Figure 14. Original images for Fig. 4D.

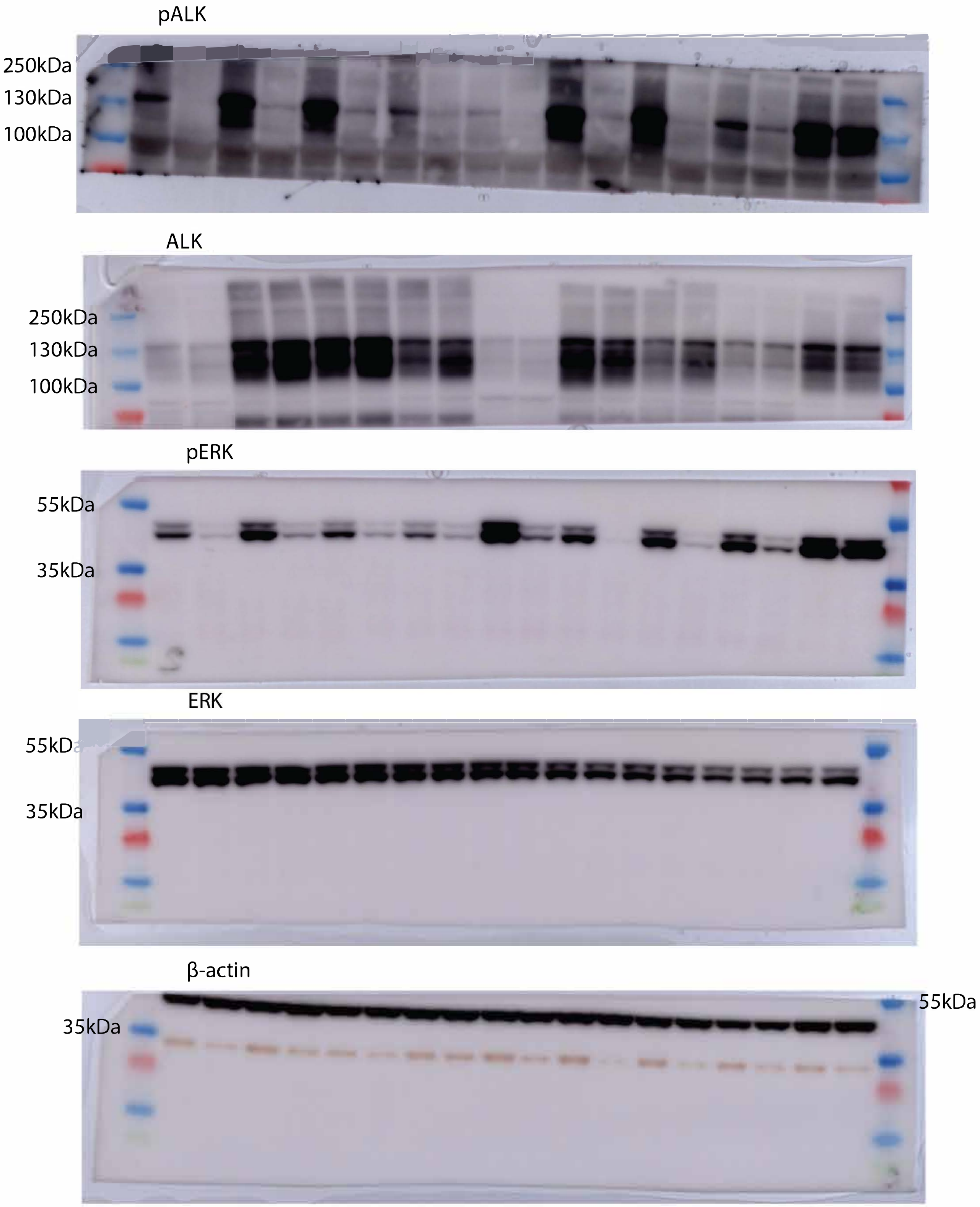

Supplemental Figure 15. Original Images for Fig. 4E.

Supplementary Tables

	Parental	erAlec	erCer	erCriz	erLor
**Criz IC50 (nM)**	59.32	589.6	765.1	552.4	756.8
**Criz bottom SE (nM)**	4.302	11.66	5.429	5.474	10.48
**Criz top SE (nM)**	3.676	4.931	3.232	3.422	4.439
**Cer IC50 (nM)**	10.36	139.9	94.15	65.26	190.2
**Cer bottom SE (nM)**	1.454	1.836	3.205	2.402	5.234
**Cer top SE (nM)**	1.958	1.167	2.761	2.07	3.253
**Alect IC50 (nM)**	4.526	81.17	126	56.17	169.5
**Alect bottom SE (nM)**	4.526	3.546	5.719	5.457	11.88
**Alect top SE (nM)**	4.572	2.688	4.891	4.739	7.435
**Lor IC50 (nM)**	1.452	4.473	14.47	13.81	4.159
**Lor bottom SE (nM)**	3.176	5.096	4.321	3.827	7.084
**Lor top SE (nM)**	5.863	7.222	4.321	3.827	18.86

Supplementary Table1. IC50 of the parental and erALK-TKI cell lines in the indicated inhibitors.

Figure	p-value
Fig. 3b Criz Week 1 vs Week 2	0.0127
Fig. 3b Criz Naïve vs Week 1	0.0162
Fig. 3b Criz Week 1 vs Week 2	0.0127
Fig. 5c ALK Criz, 3 w vs erCriz3	0.0002
Fig. 5c HER2 Criz, 3 w vs erCriz3	0.0035
Fig. 5c ALK Alec, Naïve vs 4h	0.0355
Fig. 5c ALK Alec, 3 w vs erAlec1	0.0041
Fig. 5c HER2 Alec, 3 w vs erAlec1	0.0224
Fig. 5d Criz FGFR	0.0176
Fig. 5d Criz Snai2	0.0002
Fig. 5d Alec HER2	0.0178
Fig. 5d Alec erCriz	0.0017
Fig. 5d Lor FGFR	0.0112
Fig. 5e Criz ALK vs ALK + HER2	0.0259
Fig. 6a Alectinib Week 7 vs 8	0.0005
Fig. S3A DMSO	0.0005
Fig. S3C Alectinib	0.0004
Fig. S3C Lorlatinib	0.0227
Fig. S11B Lorlatinib Subcutaneous	0.0008
Fig. S11B Lorlatinib Lung	0.0186

Supplementary Table 2. Exact p values for the indicated statistical tests.

Supplementary Methods

1. Modeling setup

We have developed a stochastic simulation to study, *in silico*, the *in vitro* cell culture experiment (**Fig. 3A**) discussed in Results Section III of the main text. We have chosen a cellular automaton framework, an individual based model in which each individual cell is tracked through time and space, and which follows simple update rules after preset time steps ($\Delta t = 16hrs$, therefore $1\ wk \approx 9\Delta t$). Each update includes randomly chosen cellular events, which include divisions, mutations and interactions with other cells in a regular grid in 2-dimensional space (illustrated in **Supplemental Methods Fig. 1**).

We chose to model each colony individually, and therefore our cellular automaton model starts with a population size of one, and in each time step, each cell is assumed to have a chance to divide if there is an empty space in its (Moore) neighborhood. If it divides, it places a daughter into its current position and a uniformly randomly chosen empty neighboring space. If there is no direct empty neighboring space, it can still divide if there is an empty spot one lattice step beyond its immediate neighborhood by 'pushing' an adjacent cell to occupy that spot to divide. However, we assumed cells cannot divide by pushing two or more layers.

There are 4 parameters involved, (i-ii) the upper and lower limits of division rates, $\boldsymbol{d_0}$ and $\boldsymbol{d_1}$, (iii) the number of mutation steps taken to reach d_1 when starting from d_0, $\boldsymbol{n_{step}}$ (illustrated in **Supplemental Methods Fig. 2**), and (iv) mutation rate, $\boldsymbol{\mu}$. The two biological hypotheses were tested using different n_{steps}: $n_{step} = 1$ for a single mutation step, and $n_{step} > 1$ for multiple steps. Then, given a specific range of division rates, $[d_0, d_1]$, the mutational increase in division rate (fitness) is $\Delta d = (d_1 - d_0)/n_{step}$ (see **Supplemental Methods Fig. 1** for a visual explanations of Δd in terms of other parameters).

Additionally, in our study, higher values of μ have been paired with scenarios with higher n_{step} values, as such scenarios require more mutations to achieve equivalent (observed) division rates, necessitating higher mutation probabilities. The rule we used to choose μ for n_{step} is $\mu_{n_{step}} = n_{step} * \mu_1$, where μ_1 is the mutation rate in the single step scenario. In this way, we model low probability (rare) mutations with huge fitness effects to high probability (common) mutations with tiny fitness effects.

The biological stochasticity is accounted for by randomly deciding the (i) spot to divide, as well as the (ii-iii) timing of division and mutation events, occurring with probability d and μ respectively. **Supplemental Methods Fig. 1** shows a detailed algorithm for this model. With the inputs being division rate ($d^{(i)}$) and mutation stage ($0 \le n^{(i)} \le n_{step}$) of a cell at the i-th time step, if the cell divides, updated $d^{(i+1)}$ and $n^{(i+1)}$ are outputs, as well as a new topology of cell spatial occupation.

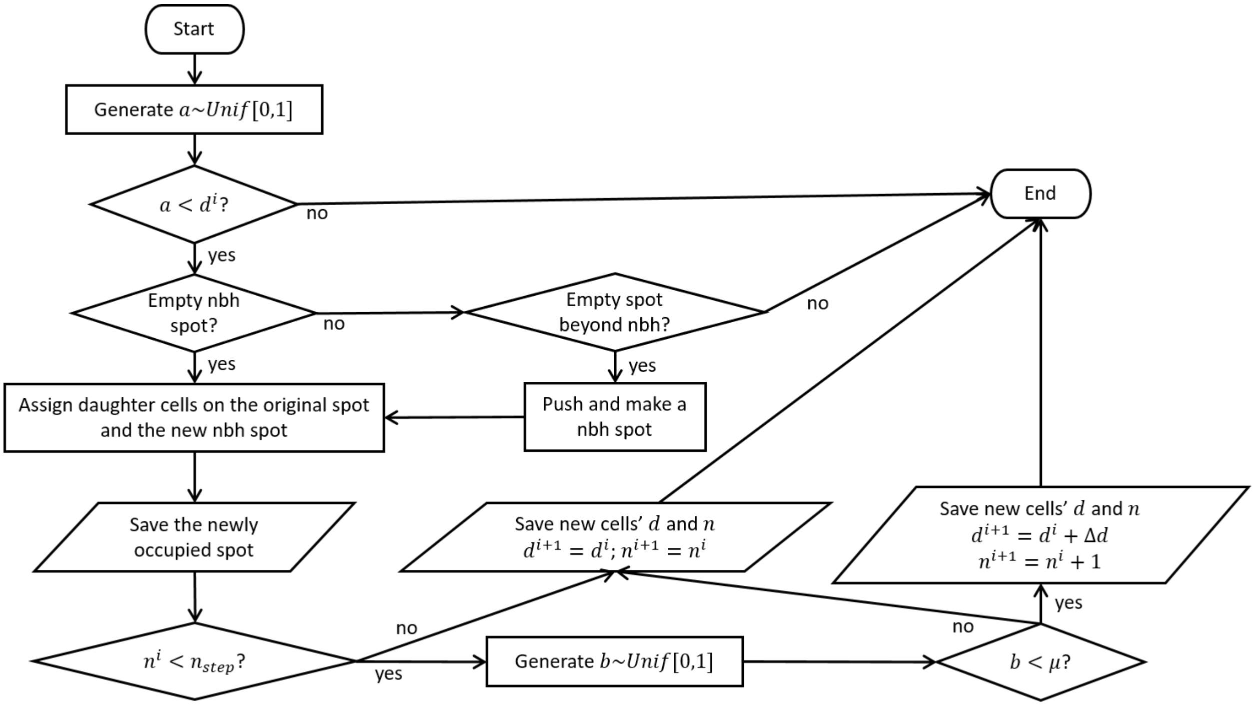

Supplementary Methods Figure 1. Algorithm to update each cell into the next time step in the cellular automaton model.

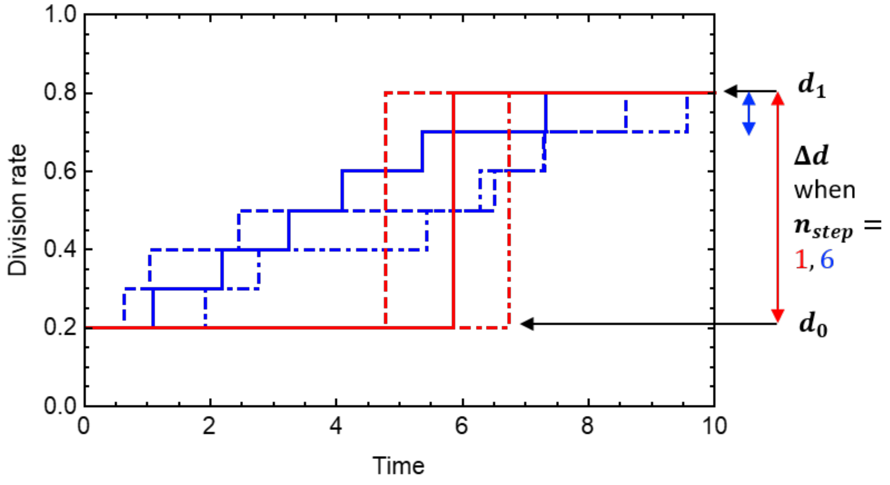

Supplementary Methods Figure 2. Illustration of the evolutionary increase in division rate, randomly increasing (by mutation) over time. With a given range of division rate, $[d_0, d_1]$, three examples of fitness histories are shown for each scenario: the single-step scenario (red curves), and the six-mutation step scenario (blue curves).

2. Parameter calibration

We calibrated model parameters in two steps.

(Step 1) calibration of range of division rates $[d_0, d_1]$:

To calibrate the initial division rate, d_0, we utilized the distribution data from the control assay colony sizes (**Fig. 3C**). Assuming only minor variance in the distribution of drug naïve cell division rates (in the presence of drug), we calibrated a single fixed value of d_0 using the best fit to the observed initial distribution. However, when calibrating the upper limit, we derived multiple values of d_1s, each of which is relevant to an individual resistant colony size. This empirical distribution of varying d_1s was used to generate diverse *in silico* colonies. Specifically, we first generated 500 *in-silico* colonies for every $d_1 \in \{0.01, 0.02, 0.03, \ldots ,0.99,1.0\}$ (with mutation not allowed for fully resistant cells, $d = d_1$), based on the algorithm described in **Supplemental Methods Fig. 1**. Then we interpolated values of d_1 and the median of 500 colony sizes in d_1, $\{d_1, \widetilde{P_{d_1}}\}$. These values were used to parameterize d_1 (**Supplemental Methods Fig. 3a**) for each colony size. **Supplemental Methods Fig. 3b** shows the distribution of d_1 derived in this way.

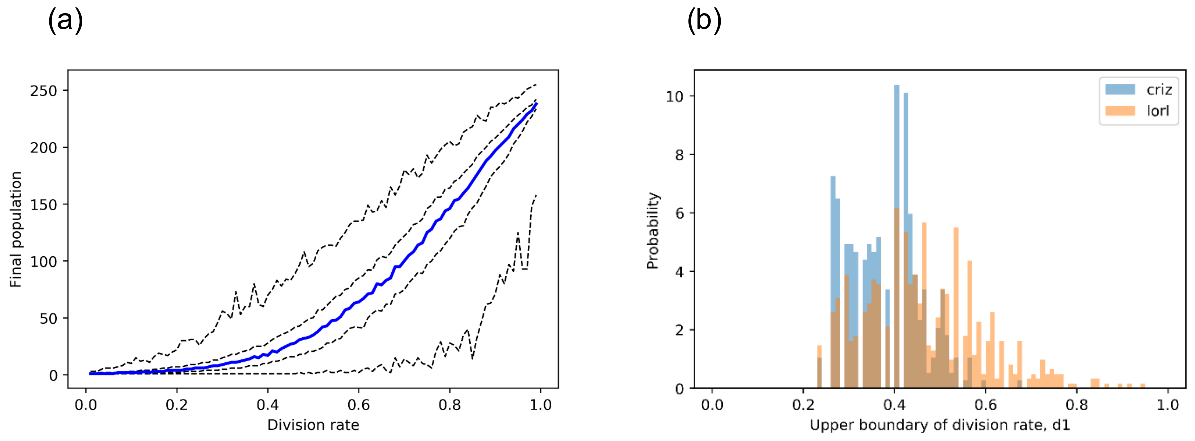

Supplementary Methods Figure 3. (a) Simulated relationship between fixed division rates and final population sizes, after growing for one week. The thick blue line represents the median and dashed lines represent 0%, 25%, 50%, 75% and 100% quantiles of 500 realizations. (b) Distributions of the upper limit of division rates, d_1, calibrated based on the median curve from (a).

Based on the relative homogeneity of drug naïve cell colony sizes, in drug, we calibrated just one fixed value for d_0. The colony sizes simulated with a fixed d_0 show a good fit to our data (see the first couple of columns in **Supplemental Methods Fig. 4**). However, the colony sizes generated with a distribution of varying d_0 (based on the median curve calibration of **Supplemental Information Fig. 3a**) does not, probably due to the right skewness of the distribution for low division rates (see the second couple of columns in **Supplemental Methods Fig. 4**). Therefore, the calibration method using various division rates seems proper only for evaluating d_1 (see the third couple of columns in **Supplemental Methods Fig. 4**).

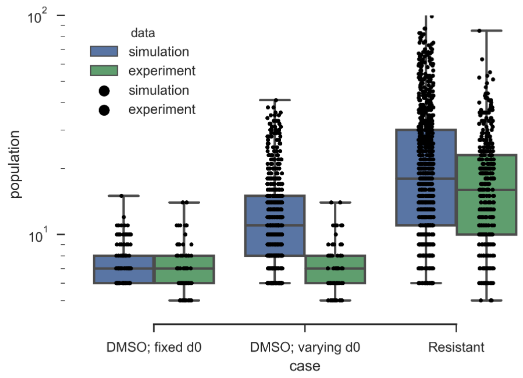

Supplementary Methods Figure 4. Comparison between experimental data and in-silico data simulated with the calibrated parameters, d_0 and d_1. Box bounds show quartiles, center shows median, error bars show minimum and maximum values.

(Step 2) calibration of mutation steps (n_{step}) and mutation rates (μ):

Based on the calibrated d_0, d_1 and the clonogenic assay data for cells with different exposure times to the drugs (i.e., 1-, 2-, 3-weeks exposure duration), we performed an exhaustive parameter sensitivity analysis for n_{step} and μ values.

For every choice of $\{n_{step}, \mu\}$, we ran 100,000 random simulations, following our stochastic algorithm, designed to mimic our experimental protocol. The simulation results in 100,000 virtual colonies of varying size when seeding cells with 1-, 2- and 3-weeks of drug exposure, as in the experimental conditions. To measure the closeness between our experimental and simulated results, we defined an error estimator between two distributions based on the Kullback–Leibler (KL) divergence. Specifically, we binned empirical observations into 10 bins based on the minimum (m) and maximum (M) values of the dataset. The lengths of the bins are all same: $\Delta b = (M - m)/9$, and the bins are half-open intervals $[m + (i-1)\Delta b, m + i\,\Delta b)$ for $i = 1, 2, \ldots, 10$. With such bin ends, we evaluated normalized bin counts for empirical data (p_i, $\sum_{i=1}^{10} p_i = 1$). Similarly, we binned the corresponding simulated data (q_i), but clipped it into the interval $[m, M]$ before binning so that the total probability equals 1 ($\sum_{i=1}^{10} q_i = 1$) (see **Supplementary Methods Fig. 5** for a visual explanation based on an example). As we have the data for three different exposure periods ($wk = 1,2,3$), we measured KL divergence for each week, and used the average of them as the error, which is expressed by:

$$KL = \frac{1}{3}\sum_{wk=1}^{3} \sum_{i=1}^{10} p_i^{wk}\, log\left(\frac{p_i^{wk}}{q_i^{wk}}\right).$$

We estimated the error over a range of $\{n_{step}, \mu\}$ parameters. See the main text for results.

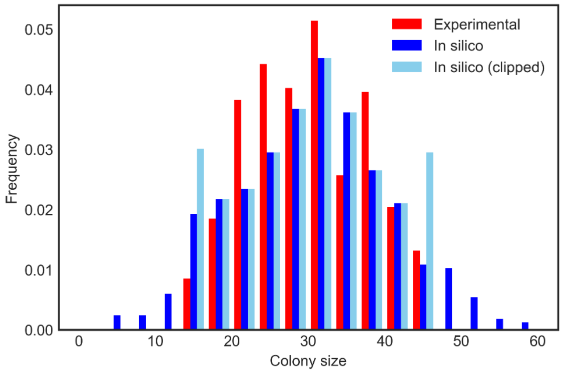

Figure 5. An example distribution of experimental data binned into 10 bins (red), and the corresponding simulated distribution (blue) clipped by the minimum and maximum of the bin ranges (sky blue).

3. Model extension by including (i) stochasticity in mutation step (Δd) and (ii) cell death

In the model described above, we accounted only for components that are indispensable when answering our primary question. In the main body of our article, we showed results based on this basic model. However, as an extension of this work, we developed extended models to account for two additional biological features, (i) variation in the influence of a mutation on fitness and/or (ii) cell death.

In one of the models, we assigned a death probability of $\delta = 0.01$ ($\approx 0.1\ d_0$). Therefore, each cell can die with a probability δ, divide with d, and remain the same otherwise (M1). In the second model, in addition to a death probability ($\delta = 0.01$), we considered bi-directional random changes in proliferation rates as a result of mutations (M2). Instead of the deterministic increase in division rate Δd, we used stochastic steps $U(\{-1,1\}) \times \mathrm{Pois}(1) \times \Delta d$, where $U(\{-1,1\})$ which follow a discrete uniform distribution drawing -1 and 1 with equal chances and arise according to a Poisson distribution with a mean of 1 timestep.

With the modified versions of the model, we carried out an identical parameter calibration and KL divergence evaluation as with earlier models. The results still support a multi-step model of resistance evolution (**Supplementary Methods Figure 4e, f**).
