# Supplementary material for: Resistance to targeted therapies as a multifactorial, gradual adaptation to inhibitor specific selective pressures
Source: Nat Commun. 2020 May 14;11:2393. doi: 10.1038/s41467-020-16212-w (PMC7224215; doi:10.1038/s41467-020-16212-w)
Supplement: Supplementary file 3 — Description of Additional Supplementary Files [file 41467_2020_16212_MOESM3_ESM.docx]

Description of Additional Supplementary Files

**Supplementary Movie 1.** Time lapse video of growth of GFP labelled NCI-H3122 cells growing in 0.5 uM lorlatinib. Images were acquired at 4x magnification every 12-24 hours.

**Supplementary Movie 2.** Visualization of in silico agent-based simulation of colony growth, reported in Fig. 3D.
